# Protein–protein interaction network analysis applied to DNA copy number profiling suggests new perspectives on the aetiology of Mayer–Rokitansky–Küster–Hauser syndrome

**DOI:** 10.1038/s41598-020-79827-5

**Published:** 2021-01-11

**Authors:** Paola Pontecorvi, Laura Bernardini, Anna Capalbo, Simona Ceccarelli, Francesca Megiorni, Enrica Vescarelli, Irene Bottillo, Nicoletta Preziosi, Maria Fabbretti, Giorgia Perniola, Pierluigi Benedetti Panici, Antonio Pizzuti, Paola Grammatico, Cinzia Marchese

**Affiliations:** 1 Department of Experimental Medicine, Sapienza Università Di Roma, Viale del Policlinico, 155, 00161 Rome, Italy; 2 Division of Medical Genetics, IRCCS Casa Sollievo Della Sofferenza Foundation, San Giovanni Rotondo, FG Italy; 3 Division of Medical Genetics, Department of Molecular Medicine, Sapienza Università di Roma, Rome, Italy; 4 Department of Maternal, Infantile and Urological Sciences, Sapienza Università di Roma, Rome, Italy

**Keywords:** Computational biology and bioinformatics, Developmental biology, Genetics, Systems biology, Diseases, Medical research, Pathogenesis, Anatomy

## Abstract

Mayer-Rokitansky-Küster-Hauser (MRKH) syndrome is a rare disease, characterised by the aplasia of vagina and uterus in women with a 46,XX karyotype. Most cases are sporadic, but familial recurrence has also been described. Herein, we investigated an Italian cohort of 36 unrelated MRKH patients to explore the presence of pathogenic copy number variations (CNVs) by array-CGH and MLPA assays. On the whole, aberrations were found in 9/36 (25%) patients. Interestingly, one patient showed a novel heterozygous microduplication at Xp22.33, not yet described in MRKH patients, containing the *PRKX* gene. Moreover, a novel duplication of a specific *SHOX* enhancer was highlighted by MLPA. To predict the potential significance of CNVs in MRKH pathogenesis, we provided a network analysis for protein-coding genes found in the altered genomic regions. Although not all of these genes taken individually showed a clear clinical significance, their combination in a computational network highlighted that the most relevant biological connections are related to the anatomical structure development. In conclusion, the results described in the present study identified novel genetic alterations and interactions that may be likely involved in MRKH phenotype determination, so adding new insights into the complex puzzle of MRKH disease.

## Introduction

Mayer-Rokitansky-Küster-Hauser syndrome is a rare disease (Online Mendelian Inheritance in Man—OMIM #277000) with an incidence of about 1 in 4500 new-born females. It is characterised by congenital aplasia of the vagina and uterus in women showing a 46,XX karyotype and normal ovarian function leading to a normal development of secondary sexual features. The condition can be classified in MRKH type I, which accounts for about 44% of cases and is characterised by isolated malformations of vagina, cervix and uterus, and in MRKH type II, accounting for about 56% of cases, in which genital anomalies are associated with renal, skeletal, cardiac and/or hearing defects (MURCS, Müllerian Renal Cervical Somite)^1–3^. MRKH syndrome is the second most common cause of primary amenorrhea after gonadal dysgenesis^4^ and it prevents patients to have normal sexual intercourses, resulting in infertility and psychological distress^5,6^. Conservative and minimally invasive surgical correction of the vaginal defect and vaginoplasty, performed with different clinical approaches, allow patients to have normal sexual functions and, possibly, reproduction with assisted techniques^7–10^. Since MRKH patients show normal ovarian function, surrogate conceiving as well as transplantation of a healthy uterus represent a chance for MRKH women to have a biological child^11–15^. Moreover, corrective interventions on a uterus with functioning endometrium may offer some patients an opportunity to have a spontaneous pregnancy^16^. To date, no publications describe the birth of a MRKH girl from a MRKH mother, however, genetic counselling is recommended to ascertain the reproductive risk of MRKH patients.

At present, the aetiology of the syndrome remains unclear; the phenotypic variability among patients, the presence of both sporadic and familial cases and the discordance between monozygotic twins suggest that MRKH syndrome is a complex disease, in which genetic, epigenetic and environmental factors are involved^17,18^. In familial cases, MRKH syndrome shows an autosomal dominant inheritance pattern, with incomplete penetrance and highly variable expressivity^19^. Genomic rearrangements and gene mutations have been reported only in a few MRKH women, whilst *in uterus* epigenetic modifications and/or environmental factors might explain the disease phenotype in patients without any genetic alteration. The mutational analysis of genes related to MRKH-associated pathologies (e.g. galactosemia) or affecting early developmental stages (*WT1, PAX2, HOXa9-13, HOXb9-13*) or mapped into the copy number variations (CNVs) recurrently found in MRKH patients (*LHX1, HNF1B, TBX6, SHOX*), has led to unclear results about putative MRKH causative mutations^3^. As concerning epigenetic modifications, Rall et al.^20^ demonstrated that DNA methylation might play a pivotal role in MRKH syndrome aetiology by modulating the expression of genes correlated to the female reproductive tract. Furthermore, a whole-genome microarray gene expression profiling, performed on vaginal vestibule tissue, showed deregulated levels of development-related genes such as *MUC1*, *HOXB2*, *HOXB5*, *HOXC8*, *JAG1* and *DLL1* in MRKH patients in comparison to healthy women^21^.

Copy number variations (CNVs) are unbalanced rearrangements larger than 50 bp and arise from genomic instability^22^. The development of the array-based comparative genomic hybridization (array-CGH) technology has considerably improved and catalysed the detection and characterisation of CNVs in MRKH patients. Moreover, Multiplex Ligation-dependent Probe Amplification (MLPA) assays have been employed to search and further define specific gene alterations associated with the syndrome^23^. Microdeletions and microduplications at critical chromosomal regions, encompassing genes coding for proteins mainly involved in the development of female genital tract and kidneys, have been reported. Specifically, five chromosome imbalances are recurrently found in MRKH women: 1q21.1del/dup, 16p11.2del, 17q12del, 22q11.21del/dup, and Xp22dup^3,24^. Aside from CNVs frequently encountered in patients with MRKH syndrome, many chromosomal rearrangements are novel or particularly rare, making uncertain their clinical interpretation. Since these imbalances usually involve large genomic regions, including several genes with different functions, a bioinformatic analysis may be helpful in explaining the potential role of the observed CNVs. In recent years, system biology approaches have emerged as powerful tools to study complex diseases. In this field, previous knowledge of physical or functional interactions among molecules is employed to build an interaction network, that shows connections between individual nodes and at the same time brings to light higher level organization of cellular communication^25^. With the purpose of providing a more comprehensive picture underlying the pathogenic mechanisms of MRKH syndrome, the main goals of the present study are:

- to identify the presence of known and new genomic imbalances in an Italian cohort of 36 unrelated MRKH women by array-CGH and MLPA assays;

- to depict a network of protein-coding genes located into the identified CNVs and to include them in potentially MRKH-related pathways through in silico protein–protein interaction (PPI) analyses.

## Methods

### Patients

A total of 36 unrelated patients with MRKH syndrome (24 type I and 12 type II) were enrolled in this multi-centric study, involving Sapienza University (Dep.t of Maternal, Infantile and Urological Sciences, Dep.t of Experimental Medicine, Dep.t of Molecular Medicine) and CSS-Mendel Institute of Rome, Italy. Patients’ mean age at diagnosis was 22 years old (range 14–57 years old). Patients’ parents, when available, were also enrolled in order to establish the familial segregation of the detected rearrangements. Diagnostic criteria for MRKH syndrome included normal external genitalia, normal 46,XX karyotype, a primary amenorrhea, presence of pubic and axillary hair and congenital absence of vagina along with the presence of Müllerian remnants. Diagnosis of MRKH syndrome was established following standard clinical procedures as previously described by Nodale et al.^21^. No evidence of cognitive dysfunction was found in MRKH women.

### DNA preparation

For each patient, genomic DNA was extracted from peripheral leukocytes in 2 ml of an EDTA blood sample using the Genomic DNA Mini Kit (Geneaid, New Taipei City, Taiwan), according to the manufacturer’s instructions. DNA concentration and purity were determined using a NanoDrop 2000c spectrophotometer (Thermo Fisher Scientific, Waltham, MA, USA).

### Array-comparative genomic hybridisation (CGH) analysis

Genomic screening for copy number variations (CNVs) was performed using a 75 Kb resolution Human Genome CGH Microarray (180KX4; Agilent Technologies, Santa Clara, CA, USA), following the manufacturer’s protocol. Female genomic DNA (Agilent Technologies) was used as reference. Microarray data were analysed by CytoGenomics software (v 5.0.2.5; algorithm ADM-2, release hg19). CNVs recurrence and gene content were investigated by mining the Database of Genomic Variants (DGV, http://projects.tcag.ca/variation/) and UCSC Genome Bioinformatics (http://genome.ucsc.edu/index.html). CNVs were classified following the American College of Medical Genetics standards and guidelines^26^ and those seen as benign based on DGV database were not further considered. CNVs were confirmed by MLPA (see below) and/or Real-Time quantitative PCR (qPCR) performed with DNA-binding dye SYBR Green (Invitrogen Corporation, Carlsbad, CA) on ABI 7900 Sequence Detection System (Applied Biosystems, Foster City, CA), using *TERT* as a reference gene. Copy numbers were evaluated through the 2^ΔΔCt^ method^27^.

### Multiplex ligation-dependent probe amplification (MLPA) analysis

The presence of deletions and duplications in MRKH candidate genes *TBX6*, *LHX1*, *HNF1B*, *TBX1* and *SHOX* were investigated and/or confirmed by using the P463-A1 and P018-G2 MLPA kits (MRC Holland, Amsterdam, Netherlands). MLPA analysis was performed in accordance with the manufacturer’s instructions. DNA samples from 3 healthy individuals were employed as reference. The results were analysed using the Coffalyser.net software (MRC Holland) and validated by qPCR.

### PPI network analysis

System biology analysis was conducted using the STRING 9.1 database (version 11.0; http://string-db.org/)^28,29^ to combine both known and predicted protein–protein interactions in a CNV-related MRKH network. This tool generates direct (physical) and indirect (functional) associations among proteins, cross-correlating multiple datasets based on genomic context, co-expression, high-throughput experiments, and previous knowledge. The protein-coding genes found in all CNVs regions detected in our cohort (n = 63) and candidate genes from the literature (n = 33) were used as input. The interactions were obtained with a confidence score of 0.150 and considering the following parameters: experiments, databases, co-expression, neighbourhood, gene fusion, co-occurrence, and excluding interactions based on text mining. The network created by STRING 9.1 was analysed by using the Cytoscape 3.8.0 platform^30^. The additional software plugins employed in this study were CentiScaPe 2.2^31^, MCODE^32^ and stringApp^33^. CentiScaPe calculates the centrality indexes as degree (proteins with high degree are interacting with several other signalling proteins, thus suggesting regulatory “hubs”), betweenness (high values indicate the capability of a protein to bring in communication distant proteins), and stress (the higher the value the higher the relevance of the protein in connecting regulatory molecules). MCODE identifies functional clusters (highly interconnected regions) in a network. stringApp tools were employed to extrapolate aspects of the biological role and pathways associated with specific expression profiles of our CNV-related MRKH network. The yFiles Layout Algorithms plugin was used to topologically arrange the network (yWorks GmbH, Tübingen, Germany).

### Ethics approval and consent to participate

The present study was in compliance with the guidelines of the 1975 Declaration of Helsinki, as revised in 2008, and was approved by the Institutional Review Board of the Dep.t of Gynaecologic-Obstetrical and Urologic Sciences of Sapienza University of Rome (date of approval: 9/07/2015. Prot.2364/15). Written informed consent was obtained from all subjects prior to inclusion in the study. For MRKH patients under the age of 18 years, written informed consent was obtained from parents or legal guardians.

## Results

### Identification of CNVs in MRKH patients through array-CGH analysis

In the whole MRKH cohort, a total of 11 distinct genomic rearrangements were detected by array-CGH analysis in 8 unrelated individuals. We identified two imbalances in chromosomal regions already associated with MRKH syndrome: a deletion of 1410.6 Kb at chromosome 17q12 (Patient 36) and a deletion of 546 Kb at chromosome 16p11.2 (Patient 39). Specifically, Patient 36 presented congenital absence of vagina and rudimentary uterus didelphys, but no additional syndromic features, leading to a diagnosis of MRKH type I. Family history was unremarkable. Array-CGH analysis performed on mother’s DNA did not reveal any chromosomal microdeletion, whilst father’s DNA was not available for the genetic test. Concerning Patient 39, she was diagnosed with MRKH type II, showing hearing impairment and bilateral cutaneous syndactyly of the foot in addition to congenital vaginal aplasia and two rudimentary hemi-uteri. No renal, genital or skeletal defects in the proband’s family were reported. We searched for the same 16p11.2 deletion in patient’s mother but array-CGH results were negative. Father’s DNA was not available for the genetic analysis.

**Figure 1:**
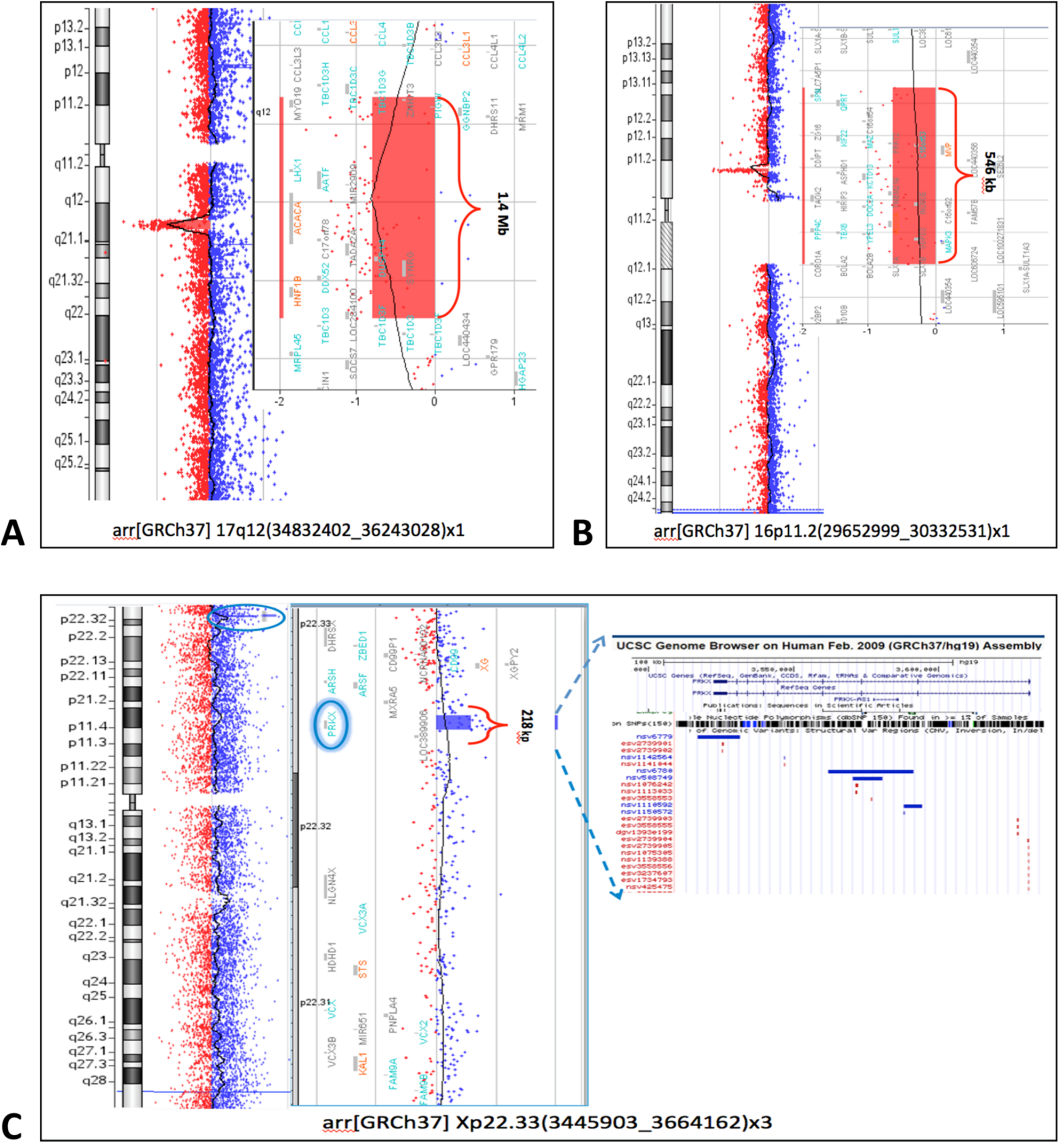
Array-CGH analysis (75 Kb average resolution). Profiles of chromosomes and details of CNVs generated by CytoGenomics (v5.0; Agilent) and showing **(A)** the microdeletion of about 1.4 Mb at 17q12 chromosome region, including *LHX1* and *HNFB1* candidate genes **(B)** the microdeletion of about 500 Kb in size at 16p11.2 chromosome region, encompassing *TBX6* candidate gene **(C)** the microduplication of about 218 Kb at Xp22.33 chromosome region, including the new candidate-gene *PRKX* (circled).

Our study also highlighted an interesting CNV not yet described in MRKH patients: Xp22.33 chromosome duplication in Patient 56. She came to our observation at the age of 57 with a clinical diagnosis of MRKH type II outlined by utero-vaginal aplasia, horseshoe kidney, mild hearing impairment, tachycardia and osteoporosis. Proband’s family history was noteworthy: nephew from her fourth brother suffers from Noonan syndrome (OMIM #163950), which is characterised by typical facial dysmorphic traits, congenital heart disease, renal malformations, developmental or behavioural problems and hearing loss; Patient 56 also referred hearing impairment for her maternal grandmother and aunt. Array-CGH analysis disclosed a microduplication (chrX:3445903–3664162;hg19) at chromosome Xp22.33. This region includes the *PRKX* gene, which encodes for a serine/threonine kinase involved in the regulation of renal epithelium morphogenesis^34^. Unfortunately, we were not able to extend the genetic analysis to both Patients 56’s parents. Array-CGH results for Patients 36, 39 and 56 are illustrated in Fig. 1A–C.

Moreover, 8 novel CNVs not yet associated to MRKH syndrome were identified in 5 cases and they were classified as CNVs with uncertain clinical significance. These CNVs, confirmed by qPCR analysis, are listed in Table 1.

**Table 1:** CNVs found by array-CGH analysis in the whole cohort of MRKH patients.

Patient	MRKH type	Phenotype	Chromosome aberration (hg19)	Size	Genes*	Matching CNV syndromes	Validation technique
**36**	I	Vaginal agenesis and two rudimentary hemi-uteri	**del17q12 (34,832,402–36,243,028)**	1410.6 kb	***PIGW***, *GGNBP2, LHX1, AATF*, ***ACACA***, *DUSP14, DDX52*, ***HNF1B, ZNHIT3***, *MYO19, DHRS11, MRM1*, *MIR2909*, *C17orf78, TADA2A, SYNRG*	17q12 deletion syndrome (OMIM #614527) MRKH^3^	MLPA
**39**	II	Vaginal agenesis and two rudimentary hemi-uteri; hypoacusis; bilateral cutaneous syndactyly of the foot	**del16p11.2 (29,652,999–30,198,600)**	546 kb	*SPN, QPRT,* ***KIF22***, *MAZ, C16orf53, MVP, KCTD13, DOC2A,* ***ALDOA***, *PPP4C*, ***TBX6***, *YPEL3, MAPK3, C16orf54, ZG16*, ***PRRT2***, *CDIPT*, *LOC440356*, *SEZ6L2, ASPHD1, TMEM219, TAOK2, HIRIP3, INO80E, C16orf92, FAM57B, GDPD3,* ***CORO1A***	16p11.2 deletion syndrome (OMIM #611913) MRKH^3^	MLPA
**44**	I	Vaginal hypoplasia	**dup17q11.2 (29,011,937–29,332,336) × 3**	320.4 kb	*CRLF3, ATAD5, TEFM, ADAP2, RNF135*		qPCR
**47**	II	Vaginal agenesis and hypoplastic uterus; unilateral renal agenesis	**del4q34.2–34.3 (177,429,071–178,785,735)**	1356.7 kb	***VEGFC***, *NEIL3, AGA,* *LINCO2509*, *LINCO1098*, *LINCO1099*		qPCR
**48**			**dup5q11.2 (58,520,261–58,613,844)**	93.6 kb	*PDE4D*^§^		qPCR
			**del7q21.11 (80,123,738–80,178,924)**	55.19 kb	*GNAT3*		
	I	Vaginal hypoplasia and two rudimental hemiuteri	**del10q11.21 (45,247,685–45,349,813)**	102.1 kb	*TMEM72-AS1*		
			**dup17q25.3 (76,138,145–76,289,356)**	151.2 kb	***TMC8***, *SYNGR2, TK1, BIRC5, C17orf99, AFMID, TMEM235*, *LINCO1993*		
**54**	I	Vaginal agenesis and uterine hypoplasia	**del8q24.11 (118,031,521–118,066,117)**	34.6 kb	***SLC30A8***^***§§***^		qPCR
**56**	II	Vaginal and uterine agenesis; horseshoe kidney; hypoacusis; tachycardia; osteoporosis	**dupXp22.33 (3445903_3664162)**	218 kb	*PRKX*		MLPA, qPCR
**57**	I	Vaginal hypoplasia	**dup13q12.13 (25,933,335–26,474,938)**	541.6 kb	***ATP8A2***		qPCR

*In bold are reported OMIM disease-genes, in underline are reported non-protein coding gene.

^§^The duplication includes the first exon of NM_001197220, transcript variant 6, of the gene.

^§§^The deletion includes the first exon of NM_001172815, transcript variant 5, of the gene.

### Novel microrearrangements detection and CNVs confirmation by MLPA analysis

**Figure 2:**
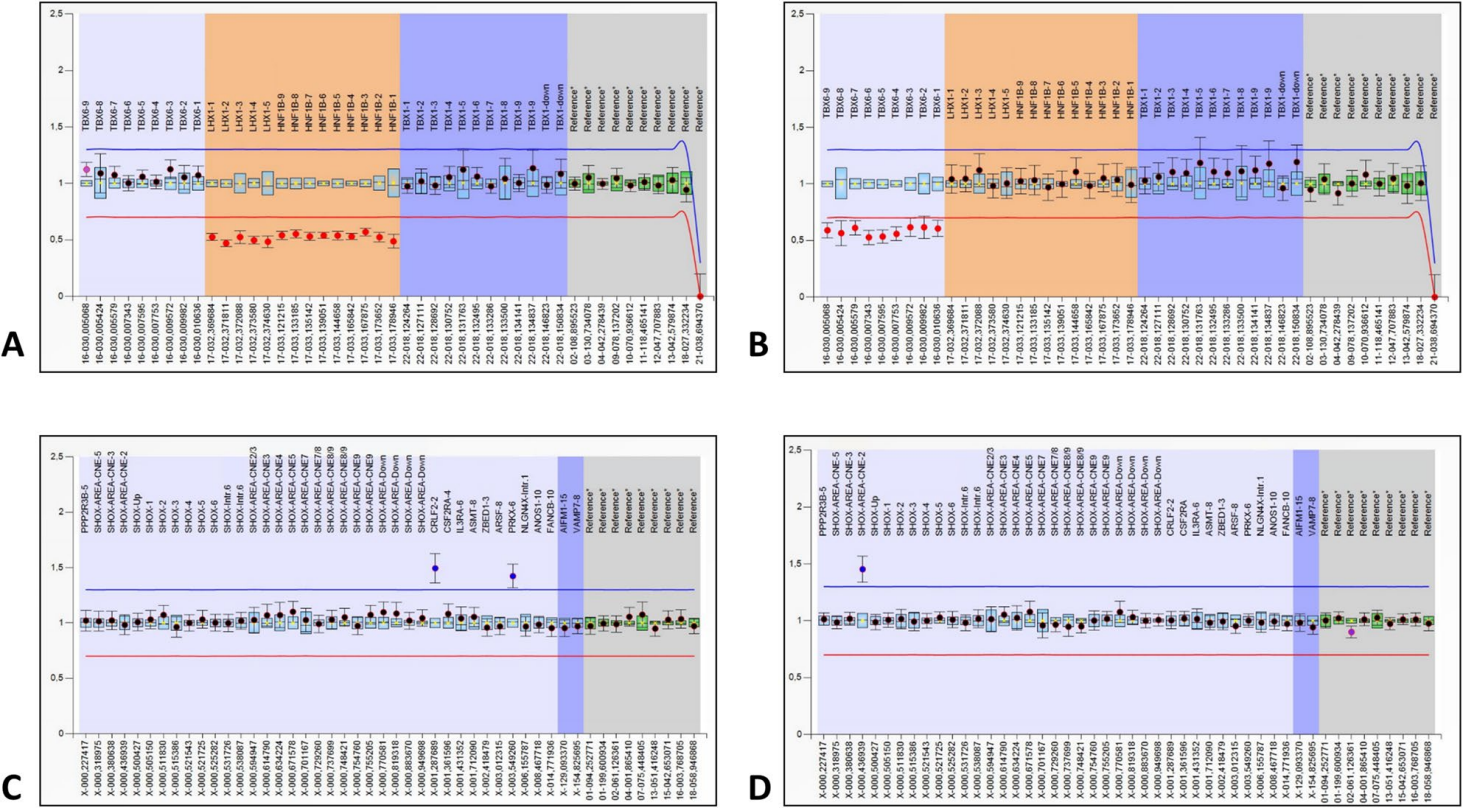
MLPA results. MLPA probes are represented on the x-axis; fluorescent intensity ratio is represented on the y-axis. Each probe ratio in a sample is represented as a dot. For each probe, the 95% confidence interval over the reference samples is depicted as a coloured bar, while the 95% confidence interval in a sample is depicted as error bars. The upper and lower arbitrary borders are shown respectively as a blue and red line. Probe ratios crossing the upper or the lower border are respectively indicative for a duplication or a deletion. **(A)** MLPA P463-A1 results of patient 36 showing the deletion of respectively *LHX1* and *HNF1B* genes. **(B)** MLPA P463-A1 results of patient 39 showing the deletion of *TBX6* gene. **(C)** MLPA P018-G2 results of patient 56 showing the duplication of *PRKX* and *CRLF2* genes **(D)** MLPA P018-G2 results of patient 64 showing the duplication of *SHOX* CNE-2 enhancer.

Locus-specific MLPA technique allowed the identification of a total of 6 genomic rearrangements. Specifically, MPLA test confirmed the alterations identified by array-CGH in Patient 36 (*LHX1* and *HNF1B* gene deletion, overlapping 17q12 chromosome deletion), in Patient 39 (*TBX6* gene deletion, in 16p11.2) and in Patient 56 (*PRKX* gene and *CRLF2* gene duplication, corresponding to Xp22.33 chromosome duplication) (Fig. 2A–C). *CRLF2* duplication has been classified as a benign CNV^35^ and was no further considered. Moreover, MLPA assay detected a novel duplication of CNE-2 *SHOX* enhancer in Patient 64 (Fig. 2D), which has not been previously associated to MRKH syndrome. However, CNVs affecting *SHOX* gene were previously related to MRKH disease^36^.

### Network analysis for protein-coding genes from identified CNVs

**Figure 3:**
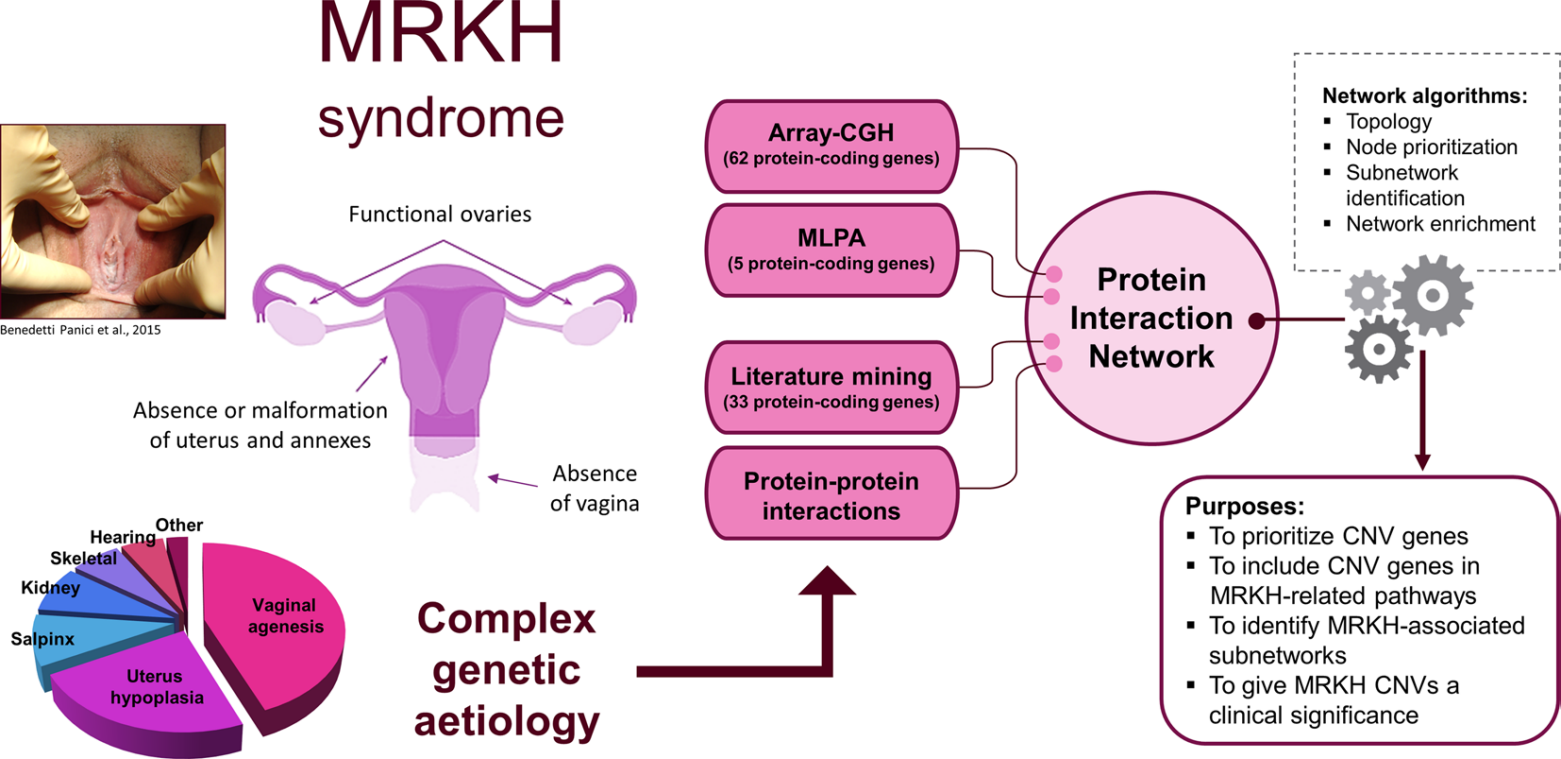
System biology analysis: aims and significance. Genetic and bioinformatic approaches combined in the study of MRKH syndrome aetiopathogenesis. Patient’s image was taken from a previously published study by our research group^10^.

**Figure 4:**
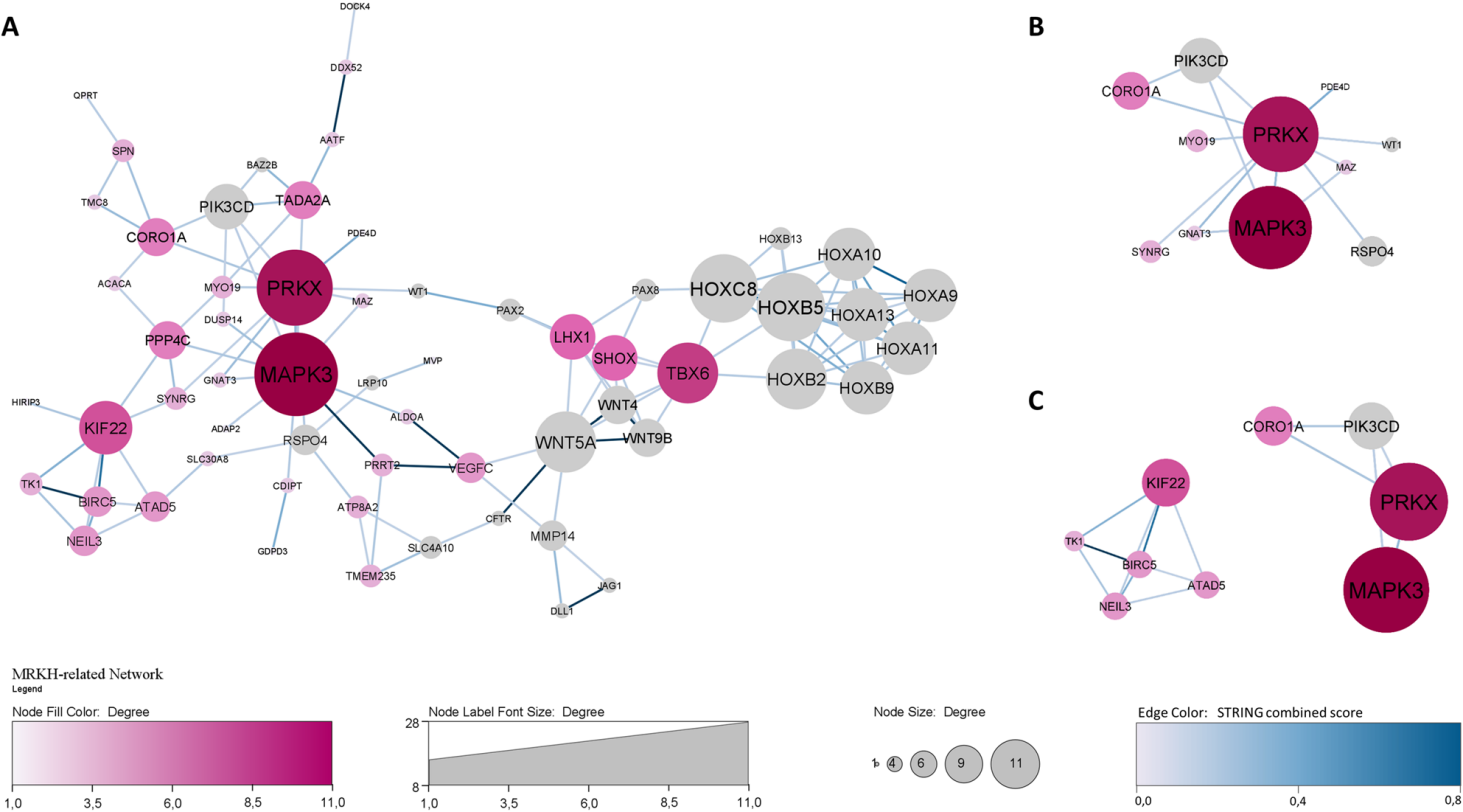
PPI network analysis. **(A)** Confidence-based network of protein–protein interactions including protein-coding genes located in the CNVs identified in the present study (purple nodes) and candidate genes from the literature (grey nodes). Nodes and font size are positively related to connectivity degree, which is further underlined by colour gradient for CNVs-related genes. Edges colour gradient is associated with STRING combined score, computed by combining the probabilities from the different evidence channels and corrected for the probability of randomly observing an interaction. **(B)** Focus on *PRKX* interactions with its first neighbours, functionally involved in movement of cells or subcellular components and cell migration (GO processes, p < 0.05). **(C)** The two most relevant clusters containing the highest percentage of CNVs-related genes, involved in cytoskeleton organization and regulation of cell migration processes.

To better understand the genetic puzzle involved in MRKH syndrome, we used a system biology approach to integrate genetic and bioinformatic data (Fig. 3). An interaction network was initially created based on 63 protein-coding genes originated from the identified CNVs by applying STRING data to Cytoscape software. Protein-coding genes not showing any interaction were discarded from the study, so we obtained one main network of 32 nodes and four side networks with only 2 nodes each. We eliminated the protein-coding genes of side networks (*TEFM, DHRS11, SYNGR2, C17orf99, TBX6, LHX1, ASPHD1, SEZ6L2*) to better calculate centrality indexes with CentiScaPe plugin, which underlined the presence of 3 central regulatory genes (*MAPK3, PRKX, KIF22*) with a connectivity degree ≥ 5. The degree is the number of links of a node with other nodes (named neighbours) in the network. Nodes with very high degree are called hubs since they are connected to many neighbours and it is likely that these hubs represent the core of a cluster. In order to demonstrate that, we used MCODE algorithm, through which we found two clusters (degree cutoff = 2), the first one including *KIF22, ATAD5, NEIL3, BIRC5, TK1* genes (score = 4.5) and the second one consisting of *MAPK3, PRKX, PPP4C, CORO1A, SPN, SYNRG, TMC8, MAZ, GNAT3* genes (score = 3). We also performed a functional enrichment analysis through stringApp, which underlined an over-representation of genes coding for molecules belonging to cell metabolism and intracellular trafficking pathways. No direct correlation between these pathways and MRKH pathogenesis was found. To improve the number of interactions and discover new connections, we added 33 candidate genes, selected from the literature, to the initial 63 genes (Table 2). We picked these 33 genes for their proved involvement in urogenital development processes, because they harboured de novo deleterious mutation in MRKH patients in trios-based studies, or because they were recurrently found as altered in MRKH cases. So, we obtained a principal network with 61 nodes and four side networks, with only 2 nodes each. We removed the protein-coding genes of side networks (*TEFM, DHRS11, SYNGR2, C17orf99, GALT, TNK2, ASPHD1, SEZ6L2*) and identified 7 hub genes (*MAPK3, PRKX, HOXB5, HOXC8, WNT5A, HOXB2, TBX6*—connectivity degree ≥ 8) (Fig. 4A,B). By MCODE analysis, we found 5 clusters. The first one (showing the highest score) included 8 nodes (*HOXC8, HOXB2, HOXB5, HOXB9, HOXa9-13*) and 27 edges. The second one included 6 nodes (*SHOX, LHX1, TBX6, WNT4, WNT5a, WNT9b*) and 14 edges. The third one consisted of 5 nodes (*KIF22, NEIL3, BIRC5, ATAD5, TK1*) and 9 edges. The fourth one contained 4 nodes (*MAPK3, PRKX, PIK3CD, CORO1A*) and 5 edges. The fifth one showed 3 nodes (*TMEM235, ATP8A, SLC4A10*) and 3 edges. We performed functional enrichment analysis through stringApp, firstly on the entire network, then on selected clusters. By filtering for Gene Ontology (GO) terms, a high number of genes (n = 35) of our network was significantly involved in anatomical structure development (p = 1.09e−5). When we focused on the highest statistical significance, GO processes linked to our network analysis were embryonic morphogenesis (p = 4.8e−9), tissue morphogenesis (p = 4.8e−9) and urogenital system development (p = 3.7e−8). The two most relevant clusters were the third one and the fourth one, both containing the highest percentage of genes from the CNVs identified in the present study (Fig. 4C). Functional enrichment analyses for the *KIF22-NEIL3-BIRC5-ATAD5-TK1* cluster and the *MAPK3-PRKX-PIK3CD-CORO1A* cluster highlighted significant correlation with cytoskeleton organization and regulation of cell migration (p < 0.005), key processes in tissue morphogenesis. The Organic Layout algorithm was applied to the network to better define its topological organization, by exposing the inherent symmetric and clustered structure of the graph, a well-balanced distribution of nodes, and few edge crossings.

**Table 2:** PPI network analysis input genes.

Protein-coding genes	Type	References
*PIGW, GGNBP2, LHX1, AATF, ACACA, DUSP14, DDX52, HNF1B, ZNHIT3, MYO19, DHRS11, MRM1, C17orf78, TADA2A, SYNRG, SPN, QPRT, KIF22, MAZ, C16orf53, MVP, KCTD13, DOC2A, ALDOA, PPP4C, TBX6, YPEL3, MAPK3, C16orf54, ZG16, PRRT2, CDIPT, SEZ6L2, ASPHD1, TMEM219, TAOK2, HIRIP3, INO80E, C16orf92, FAM57B, GDPD3, CORO1A, CRLF3, ATAD5, TEFM, ADAP2, RNF135, VEGFC, NEIL3, AGA, PDE4D, GNAT3, TMC8, SYNGR2, TK1, BIRC5, C17orf99, AFMID, TMEM235, SLC30A8, PRKX, ATP8A2, SHOX*	CNV-related (n = 63)	
*WNT4, GALT, CFTR, HOXA9, HOXA10, HOXA11, HOXA13, WNT9B, MMP14, LRP10, GREB1L, DOCK4, RSPO4, PAX8, EMX2, WNT5A, BAZ2B, KLHL18, PIK3CD, SLC4A10, TNK2, WT1, PAX2, HOXB9, HOXB10, HOXB11, HOXB13, MUC1, HOXC8, HOXB2, HOXB5, DLL1, JAG1*	Candidate genes (n = 33)	^3, 21, 69–71^

## Discussion

The genetic contribution to MRKH syndrome remains largely unknown, even if an autosomal dominant or a multifactorial pattern of inheritance are most likely and specific genetic alterations have been involved in MRKH phenotype determination^3,19,37^. In the last years, the application of array-CGH technique has allowed research groups to disclose cryptic chromosome imbalances at the basis of different congenital malformations. By using this approach, we detected 11 distinct chromosomal rearrangements in our cohort of 36 MRKH patients. Moreover, through MLPA screening further alterations involving four MRKH candidate genes were found. Table 1 summarises chromosomal rearrangements observed in our genetic analyses.

Although various CNVs have been identified in MRKH patients, their significance in this complex genetic disease remains uncertain mainly due to the lack of proof causation. Nowadays, the availability of large amounts of biomolecular information poses the challenge to arrange these data into a biological context. In particular, PPI network analysis allows narrowing a list of candidate genes to those whose products are known (or predicted) to interact with each other, thereby enriching for genes potentially contributing to a same biological process. The precondition for using this kind of data integration hinges on the assumption that proteins related to the same diseases or clinical features tend to interact or are closely located in the network. So, by building a net of 61 nodes, we succeed in linking together 36 protein-coding genes from the identified CNVs and in connecting them with pathways previously related to MRKH phenotype determination. Although not all of these genes taken individually seem to have a clinical significance, their combination in a computational network may suggest new points of view and interpretations for MRKH syndrome. Actually, the evidence that gene products interact with each other to form an intricate network indicates that a perturbation in one of these components can be propagated through their interactions, so affecting other genes in the network. The significance of central regulatory nodes is related to the fact that their removal has a great impact on the network topology. Indeed, it has been shown that biological networks tend to be robust against random perturbations, but hub disruption often leads to system failure^38^.

As reported, array-CGH detected a microdeletion at chromosome 17q12, which encompasses *LHX1* and *HNF1B* genes, in one MRKH affected women of our Italian cohort, so confirming this region as the most frequent chromosomal alteration associated with MRKH syndrome^39–43^. *LHX1* encodes a transcription factor belonging to the LIM homeodomain family, and mouse studies have shown that this gene has a main role in female reproductive tract development^44^ and renal morphogenesis^45,46^. *HNF1B*, encoding a POU homeodomain-containing transcription factor, is highly expressed in Müllerian ducts during embryogenesis and it promotes *LHX1*, *PAX2* and *WNT9B* expression, which in turn are essential for Müllerian ducts formation, elongation and maintenance^47,48^. Indeed, *HNF1B* haploinsufficiency is related to uterine and renal malformations in MRKH patients^3^. Notably, in our PPI network *LHX1* is a central node of a cluster, interacting with *WNT4* and *WNT5A*, which together with *PAX2* and *WNT9B* are main regulators of Müllerian ducts formation^48,49^. In view of these considerations, our findings confirm *LHX1* and *HFN1B* as strong candidate genes for MRKH syndrome and push towards additional functional studies to better understand their biological role in this syndrome.

Concerning 16p11.2 deletion, alterations in this chromosome region have been previously linked to MRKH disease by array-CGH studies^42^. In a patient affected by MRKH type II, we identified a chromosomal deletion including *TBX6*, a candidate gene for MRKH syndrome already described in the literature as deleted in other 9 cases of this syndrome^42,43^. *TBX6* gene encodes a T-Box transcription factor, playing a crucial role in mesoderm formation and specification during embryonic development. Studies conducted on mouse models showed that lack of *TBX6* leads to vertebral and rib anomalies together with urogenital malformations^50,51^, features often associated to MRKH syndrome. Indeed, *TBX6* is a hub in our CNV-related PPI network and its connection with *HOXB2*, *HOXB5*, *HOXC8* as well with *WNT4*, *WNT5A*, *WNT9B* and *LHX1*, all implicated in organ morphogenesis and embryo development^3,21,48^, highlights the importance of *TBX6* in MRKH aetiopathogenesis.

Genetic study on MRKH women with locus specific MLPA assays has also revealed the presence of a duplication involving *SHOX* gene in one patient. *HOX* genes encode for highly conserved transcriptional and epigenetic factors that are master regulators of the embryonic development at different times and in specific body axis regions; they also maintain their expression throughout postnatal life^52,53^. *SHOX* gene, which is located in the PAR1 region of the X and Y chromosomes, is specifically involved in the skeletal development. Here we report a novel duplication of a specific *SHOX* enhancer (i.e. CNE-2) highlighted by MLPA analysis. This genetic alteration has not been described before in the literature. Heterozygous duplications partially including *SHOX* have been previously described in both familial and sporadic MRKH cases, all exhibiting type I-associated clinical characteristics^35,36^. The current hypothesis is that a duplication involving *SHOX* or its enhancers might determine a gain of function effect leading to a wide dysregulation of gene expression in early steps of the female genital tract development. It is interesting to note that *SHOX* is a central protein-coding gene in one of the clusters of our MRKH-related network, interacting with *PAX2*, *WNT4*, *WNT5A* and *WNT9B* as well as *TBX6*.

The search for genetic anomalies through array-CGH highlighted a microduplication at chromosome Xp22.33. Although this specific region has already been described as rearranged in MRKH cases^41^, the portion that we found altered is unique and it represents the most intriguing finding of the present study. Indeed, the Xp22.33 region contains the *PRKX* gene, which encodes for a serine/threonine kinase, with a known role in renal epithelium morphogenesis^34,54–56^ but not yet associated with Müllerian ducts’ anomalies. Li et al.^57^, by using a cell line derived from normal human foetal collecting tubule epithelia, demonstrated that *PRKX* expression activates cAMP-dependent renal epithelial cell migration and tubular morphogenesis. The same research team also showed that *PRKX* expression stimulates ureteric bud branching and the induction of glomeruli in an embryonic kidney organ culture system^58^. To date, mechanisms involving PRKX protein in the specific development of genital structures have not been demonstrated in vivo^59^, but we cannot exclude that this serine/threonine kinase might have a role in Müllerian ducts morphogenesis. Indeed, the formation of the different organs of the urinary and genital/reproductive systems is strongly interconnected^60^ and many common genes have been implicated in their morphogenesis^61,62^. Noteworthy, we found PRKX to be a hub in our MRKH-related network, interacting with proteins functionally involved in cell movement and migration, further supporting a potential role for this gene in MRKH syndrome determination.

With respect to that, we evaluated *PRKX* gene expression levels on vaginal vestibule tissues from selected MRKH women of our cohort. From 2006 to 2016, in collaboration with the gynaecology team of Dep.t of Maternal, Infantile and Urological Sciences (Policlinico Umberto I, Sapienza University of Rome, Italy), we performed vaginoplasty using autologous in vitro cultured vaginal tissue in 39 patients with MRKH syndrome^10,63–66^. During that time, we were able to collect RNA samples which we analysed by quantitative RT-PCR highlighting a moderate but significant increase of *PRKX* mRNA levels in 67% of patients compared to healthy controls (see Supplementary Data). *PRKX* gene expression study was achieved on adult tissues. We are aware of the fact that results obtained through this approach might not reflect *PRKX* expression yield during embryogenesis. In addition, the vaginal vestibule is not of mesodermal (or Müllerian) origin, but it derives from ectoderm, which makes rudimentary uterine tissue more indicated for this kind of analysis. Anyway, vaginal vestibule tissue taken from patients during the vaginoplasty process seems to be the least invasive procedure. Indeed, reducing psychological and physical distress in MRKH patients is one of the main goals of our medical and research approach. Additionally, we confirmed the importance of further analyses on *PRKX* role in MRKH aetiopathogenesis by interrogating public available MRKH transcriptome database^67^ (see Supplementary Data).

Concerning the 27 identified CNV genes, which were excluded from the in silico analysis, we speculate that they were not integrated in the predicted network by considering both the functional redundancy of some encoded proteins as well as the normal variation nature of many CNVs across the genome^68^. Still, it should be stressed that a network study only generates predictions, so caution is mandatory when drawing conclusion and in vitro*/*in vivo validations should always be performed.

In conclusion, our genetic and bioinformatic results give new insight in the complex gene–gene interactions in MRKH syndrome. Not least, unravelling the genetic basis of MRKH disease represents an important goal not only with respect to merely anatomical and reproductive issues, but also in pursuing psychological well-being of affected patients and their relatives. Indeed, the diagnosis of the syndrome often occurs during adolescence, a delicate time for young women. The indication of a precise genetic cause for their disorder may help MRKH patients and their family in overcoming the initial dismay and guide them in choosing appropriate clinical interventions and future reproductive choices, also considering the recurrence risk of the disease.

## Supplementary Information

Supplementary Information.

## Data Availability

Data supporting the findings of this study are available from the corresponding author upon reasonable request.
