# Supplementary material for: Protein–protein interaction network analysis applied to DNA copy number profiling suggests new perspectives on the aetiology of Mayer–Rokitansky–Küster–Hauser syndrome
Source: Sci Rep. 2021 Jan 11;11:448. doi: 10.1038/s41598-020-79827-5 (PMC7801512; doi:10.1038/s41598-020-79827-5)
Supplement: Supplementary file 1 — Supplementary Information. [file 41598_2020_79827_MOESM1_ESM.pdf]

Title

Protein-Protein Interaction network analysis applied to DNA copy number profiling suggests new perspectives on the aetiology of Mayer-Rokitansky-Küster-Hauser syndrome

Authors

Paola Pontecorvi[1], Laura Bernardini[2], Anna Capalbo[2], Simona Ceccarelli[1], Francesca Megiorni[1], Enrica Vescarelli[1], Irene Bottillo[3], Nicoletta Preziosi[3], Maria Fabbretti[2], Giorgia Perniola[4], Pierluigi Benedetti Panici[4], Antonio Pizzuti[1,2], Paola Grammatico[3] and Cinzia Marchese[1]

Affiliations

[1]Department of Experimental Medicine, Sapienza Università di Roma, Rome, Italy

[2]Division of Medical Genetics, IRCCS Casa Sollievo della Sofferenza Foundation, San Giovanni Rotondo, FG, Italy

[3]Division of Medical Genetics, Department of Molecular Medicine, Sapienza Università di Roma, Rome, Italy

[4]Department of Maternal, Infantile and Urological Sciences, Sapienza Università di Roma, Rome, Italy

SUPPLEMENTARY INFORMATION

METHODS

qRT-PCR analysis on vaginal mucosa cells samples

Primary cultures of human vaginal mucosa cells were established from 1 cm^2 full-thickness mucosal biopsy of the vaginal vestibule of 9 MRKH patients and vaginal tissue of 4 healthy control women. Following enzymatic dissociation, cells were seeded onto collagen IV (10 mg/ml)-coated culture plates and maintained in chemical defined Keratinocyte Growth Medium (KGM; Lonza Milano S.r.l., Milan, Italy). Medium was changed twice a week. Cell cultures were characterized by immunofluorescence and western blot analysis and their morphology was evaluated with a phase contrast microscopy. Expression of specific epithelial markers (K14 and K19) and lack of vimentin confirmed their epithelial origin, as previously reported [65]. Total RNA from vaginal mucosa cell cultures was extracted using TRIzol reagent (Invitrogen, Milan, Italy), following the manufacturer's instructions. RNA samples were quantified using a NanoDrop 2000c spectrophotometer (Thermo Fisher Scientific) and evaluated for degradation by agarose gel running. Total RNA (1-2 µg) was reverse transcribed using the High Capacity RNA to cDNA Kit (Applied Biosystems by Thermo Fisher Scientific) according to the manufacturer's instructions. PRKX expression

was evaluated by qRT-PCR experiments with a specific TaqMan Gene Expression Assay (PRKX, Hs_00746337_s1 by Applied Biosystems). Cyclophilin-A mRNA was used as endogenous control (PPIA, Hs_04194521_s1 by Applied Biosystems). Specimens from 4 healthy women (mean age 35 years old: range 25-54 years old) were used as reference after accurate evaluation of individual variation of gene expression. Samples were run in triplicate using an ABI 7500 Real Time instrument (Applied Biosystems), in at least two independent experiments. PRKX relative expression in MRKH patients in comparison to healthy controls was calculated by using the $2^{\Delta\Delta Ct}$ method [27]. Statistical significance of qRT-PCR data was evaluated by Student t-test using Prism 7 software. P values less than 0.05 were considered statistically significant.

RESULTS

PRKX gene expression analysis in MRKH patients

Since *PRKX* gene might be involved in MRKH, we decided to assess its expression at mRNA level in 9 MRKH patients in comparison with a pool of 4 healthy control women. The difficulty of obtaining vaginal biopsies from healthy young women accounts for the low number of controls. In order to investigate *PRKX* mRNA levels, we performed qRT-PCR experiments on RNA extracted from vaginal vestibule keratinocytes by using specific TaqMan *PRKX* primers/probe. As illustrated in Supplementary Fig. 1, *PRKX* expression showed a moderate but significant increase in 6 out of 9 MRKH patients compared to the pooled control (ranging from 1.5 to 2-fold increase; $p<0.05$). Only in Patient 40, *PRKX* expression was significantly decreased compared to the control group ($p<0.05$). Due to the small number of patients, it was not possible to correlate *PRKX* mRNA levels with syndrome type (I or II). Moreover, RNA from the vaginal mucosa of Patient 56 was not available, so we cannot directly correlate the *PRKX* chromosomal microduplication at Xp22.33 with its transcriptional overexpression. Concerning the other MRKH patients showing *PRKX* upregulation, we speculate that point mutations at *PRKX* locus or epigenetic modifications may explain the obtained qRT-PCR data.

Database interrogation suggests further analyses on PRKX

One of the obstacles in identifying molecular mechanisms of the MRKH syndrome is the lack of publicly available genomic and transcriptomic datasets for MRKH patients. Hentrich et al. assembled a large and unique cohort of MRKH type I and type II patients and profiled the endometrial tissue-related transcriptome by using RNA-seq, providing a view of the altered transcription landscape in this complex disease [67]. Moreover, they offered an online tool that allows to navigate and download these rich data from single genes to pathways (http://mrkh-

data.informatik.uni-tuebingen.de). We interrogated this database for *PRKX* gene expression, so obtaining significant results (p<0.05) about its upregulation in MRKH type II patients compared to controls. However, the p adjusted value (BH correction) seems to contradict the finding, but considering that the database is currently under development, further analyses starting from raw data are needed to be taken into account.

SUPPLEMENTARY FIGURE 1

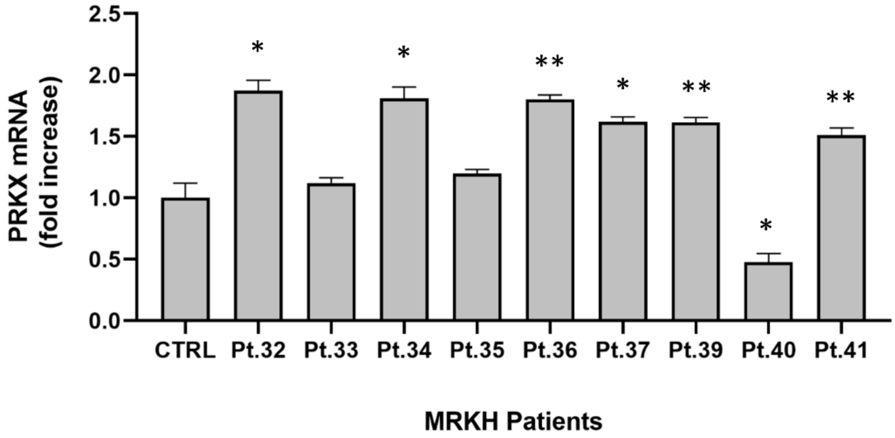

Fig. S1 *PRKX* expression analysis. qRT-PCR analysis performed on RNA from vaginal vestibule keratinocytes showed a moderate but significant increase in 6 out of 9 MRKH patients and a significant decrease in one MRKH patient compared to controls (mean ± SEM; n = 2; *p<0,05, **p<0,005; Student T test).
